# BRAF-activated long non-coding RNA contributes to cell proliferation and activates autophagy in papillary thyroid carcinoma

**DOI:** 10.3892/ol.2014.2487

**Published:** 2014-08-28

**Authors:** YONG WANG, QINHAO GUO, YAN ZHAO, JIEJING CHEN, SHUWEI WANG, JUN HU, YUEMING SUN

**Affiliations:** 1 Department of General Surgery, The First Affiliated Hospital of Nanjing Medical University, Nanjing, Jiangsu 210000, P.R. China; 2 Department of Obstetrics and Gynecology, Northern Jiangsu Province Hospital, Clinical Medical College, Yangzhou University, Yangzhou, Jiangsu 225001, P.R. China

**Keywords:** long non-coding RNA, BRAF-activated long non-coding RNA, papillary thyroid carcinoma, autophagy

## Abstract

Long non-coding RNAs (lncRNAs) are novel regulators in cancer biology. BRAF-activated lncRNA (BANCR) is overexpressed in melanoma and has a potential functional role in melanoma cell migration. However, little is known about the role of BANCR in the development of papillary thyroid carcinoma (PTC). In the present study, BANCR expression was examined in six pairs of PTC and matched adjacent normal tissues. The results revealed that BANCR levels were significantly higher in the PTC tissues and PTC IHH-4 cells compared with the normal controls. Knockdown of BANCR in the IHH-4 cells inhibited proliferation and increased apoptosis of the cells *in vitro*. Further investigation of the underlying mechanisms revealed that BANCR markedly activated autophagy. Overexpression of BANCR inhibited apoptosis in the IHH-4 cells, whereas inhibition of autophagy stimulated apoptosis in the BANCR-overexpressed cells. BANCR overexpression also increased cell proliferation and the inhibition of autophagy abrogated BANCR overexpression-induced cell proliferation. In addition, the overexpression of BANCR resulted in an increase in the ratio of LC3-II/LC3-I, a marker for autophagy, while the knockdown of BANCR decreased the ratio of LC3-II/LC3-I. These results revealed that BANCR expression levels are upregulated in PTC. Additionally, BANCR increases PTC cell proliferation, which could activate autophagy.

## Introduction

Papillary thyroid carcinoma (PTC) is the most common type of thyroid cancer, accounting for ~80% of all thyroid cancers. With appropriate treatment, including surgery and radioiodine ablation, the majority of PTCs have excellent prognoses (1). Examining genetic factors could aid early detection and also facilitate the treatment and prevention of PTC. However, the ideal genetic marker for PTC detection has not yet been identified.

Although originally considered to be spurious transcriptional noise, long non-coding RNAs (lncRNAs) are now recognized as regulators in tumorigenesis and tumor progression (2,3). Functional lncRNAs have the potential to be used for diagnosing cancer and determining prognosis, as well as being a potential therapeutic target that could become a valuable novel diagnostic and therapeutic tool (4). BRAF-activated lncRNA (BANCR) is a 693-bp transcript on chromosome 9, which is frequently overexpressed and has a possible functional role in the migration of melanoma cells (5,6). BANCR is strongly linked with ^V600E^BRAF, which is the most prevalent mutation of the BRAF gene. ^V600E^BRAF mutations are exhibited in 70% of malignant melanomas, 36–53% of papillary thyroid cancers and 5–22% of CRCs (7). The ^V600E^BRAF mutation is considered to be a putative prognostic marker for the aggressiveness of PTC (8), but the expression pattern and biological functions of BANCR in PTC remain to be elucidated.

Autophagy is a lysosome-mediated intracellular catabolic process by which cells remove damaged organelles and long-lived proteins to maintain cellular homeostasis (9). Autophagy is activated in cancer cells and contributes to tumor cell survival (10). High oncogenic BRAF levels have been shown to initiate autophagy, which is possibly involved in tumor progression (11). Since a close association exists between the presence of the BRAF gene and autophagy, it has been speculated that BANCR could be involved in the regulation of autophagy.

The aims of the present study were to detect the expression levels of BANCR and to investigate the function and molecular mechanisms of BANCR in PTC.

## Materials and methods

### Tissue samples and cell culture

In total, six specimens of human PTC and adjacent normal tissues were obtained, with informed consent, from surgeries performed between March and June 2013 at the First Affiliated Hospital of Nanjing Medical University (Nanjing, Jiangsu, China). The protocol used in this study was approved by the hospital’s Protection of Human Ethics Committee. The diagnosis of PTC was histopathologically confirmed. The resected tissue samples were immediately frozen in liquid nitrogen and stored at −80°C until RNA extraction. The human PTC-derived cell line, IHH-4, was provided by Professor Congyou Lu (The Chinese University of Hong Kong, Hong Kong, China). The IHH-4 cells were routinely cultured at 37°C in RPMI 1640 medium (Wisent, Inc., QC, Canada) with 10% fetal bovine serum (Wisent, Inc.) and 5% carbon dioxide.

### Quantitative polymerase chain reaction (PCR)

Total RNA from tissues and cells was extracted using RNAiso Plus (Takara Biotechnology (Dalian) Co., Ltd., Dalian, China), and reverse transcription (RT) reactions were performed using a PrimeScript RT reagent kit (Takara Biotechnology (Dalian) Co., Ltd.) according to the manufacturer’s instructions. Quantitative PCR reactions were prepared at a final volume of 20 μl using a standard protocol and the SYBR Green PCR kit (Roche Diagnostics Co., Indianapolis, IN, USA), and the reactions were performed on the StepOnePlus Real-Time PCR System (Applied Biosystems, Inc., CA, USA). Each reaction was performed in triplicate. The 2^−ΔΔCT^ method was used to determine the relative gene expression levels, using β-actin as the endogenous control to normalize the data. The primers used in this study were as follows: BANCR forward, 5′-ACAGGACTCCATGGCAAACG-3′ and reverse, 5′-ATGAAGAAAGCCTGGTGCAGT-3′; β-actin forward, 5′-AGAAAATCTGGCACCAACC-3′ and reverse, 5′-TAGCACAGCCTGGATAGCAA-3′; LC3 forward, 5′-CCACACCCAAAGTCCTCACT-3′ and reverse, 5′-CAC TGCTGCTTTCCGTAACA-3′. PCR was performed at 95°C for 30 sec, 40 cycles of 95°C for 5 sec, 60°C for 31 sec, and then, for dissociation, at 95°C for 15 sec, 60°C for 1 min and 95°C for 15 sec.

### Generation of stable infected cell lines

Recombinant lentiviruses containing short hairpin (sh)RNA-323 (LV-BANCR-323, GGA GTGGCGACTATAGCAAAC), shRNA-540 (LV-BANCR-540, GGACTCCATGGCAAACGTTGT), human full-length BANCR cDNA (LV-BANCR) and a negative control (LV-NC) were purchased from GenePharma Co., Ltd. (Shanghai, China). The IHH-4 cells were infected with LV-BANCR-323, LV-BANCR-540, LV-BANCR and LV-NC (multiplicity of infection, 20). The supernatant was removed after 24 h and fresh culture medium was added to the cells. The infection efficiency was confirmed by RT-PCR at 72 h post-infection, and the cells were treated with 2 μg/ml puromycin for 2 weeks.

### Cell proliferation assays

Cell proliferation assays were performed using Cell Counting Kit-8 (CCK8; Beyotime Institute of Biotechnology, Jiangsu, China). The cells were plated in triplicate in 96-well plates at ~2×10^3^ cells per well and cultured in the growth medium. The number of cells per well was measured by the absorbance at 450 nm at the indicated time*-*points, according to the manufacturer’s instructions.

### Flow cytometric analysis

The cells (4×10^5^) were seeded in 6-well plates. After 24 h, the cells were collected and incubated with Annexin V-fluorescein isothiocyanate and 7-amino-actinomycin D (Biolegend, Inc., San Diego, CA, USA) for 15 min in the dark and apoptosis was analyzed using a flow cytometer. The cell cycle was also analyzed subsequent to propidium iodide staining for 30 min.

### Transwell migration assay

In total, 4×10^4^ cells were plated in medium without serum on a non-coated membrane in the top chamber (24-well insert; 8-mm pore size; Corning Costar; Corning, Inc., Corning, NY, USA). Medium supplemented with serum was used as a chemotactic agent in the lower chamber. The cells were incubated for 24 h, and those cells that did not migrate through the pores were removed with a cotton swab. The cells on the lower surface of the membrane were stained with crystal violet (Beijing Solarbio Science & Technology Co., Ltd., Beijing, China). The cell numbers were determined by counting the penetrating cells under a microscope (Nikon, Kobe, Japan) in random fields, with five fields per chamber. Each experiment was performed in triplicate.

### Western blot analysis

Proteins were extracted with radioimmunoprecipitation assay (Beyotime Institute of Biotechnology) and equal amounts of protein were electrophoresed on a 12 or 15% sodium dodecyl sulfate-polyacrylamide gel and subsequently transferred to polyvinylidene fluoride membranes (Millipore, Boston, MA, USA). The membranes were blocked with 5% skimmed milk in Tris-buffered saline containing 0.1% Tween-20 (TBST), at room temperature for 2 h. The membranes were incubated with the rabbit polyclonal LC3-I, LC3-II (1:1,000; Cell Signaling Technology, Inc., Danvers, MA, USA) and rabbit polyclonal GAPDH (1:10,000; Beijing Biosynthesis Biotechnology Co., Ltd., Beijing, China) primary antibodies at 4°C overnight. The membranes were then washed three times with TBST and incubated with horseradish peroxidase-conjugated secondary anti-rabbit antibody (1:5,000; Beijing Biosynthesis Biotechnology Co., Ltd.) at room temperature for 2 h. Following three washes with TBST, the membranes were developed using ECL Plus (EMD Millipore, Billerica, MA, USA), and exposed to X-ray film. GAPDH was used as an internal loading control.

### Statistical analysis

Data were analyzed using SPSS 19.0 software (SPSS, Chicago, IL, USA), and are expressed as the mean ± standard deviation of data from at least three independent experiments. The differences between the groups were analyzed using Student’s t-test, Pearson’s χ^2^-test or one-way analysis of variance, as appropriate. P<0.05 was considered to indicate a statistically significant difference.

## Results

### BANCR levels are significantly upregulated in PTC

**Figure 1:**
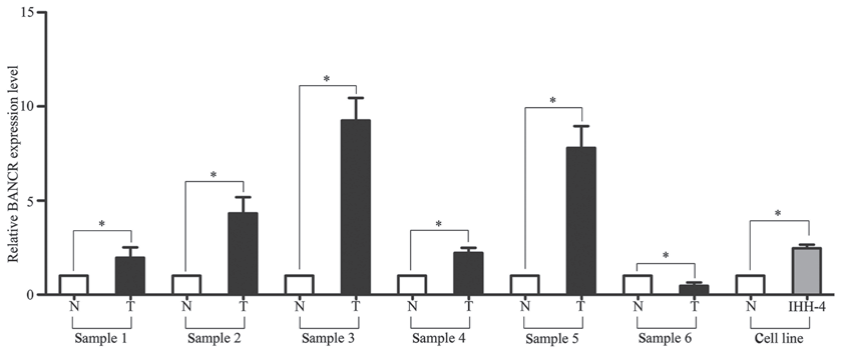
BANCR levels are upregulated in papillary thyroid carcinoma (PTC). Reverse transcription-polymerase chain reaction results showed that BANCR expression was significantly higher in five out of six of the tumor tissues compared with the adjacent normal tissues. The BANCR level in the PTC IHH-4 cell line was also upregulated compared with the mean expression level of the adjacent normal tissues. BANCR expression levels were normalized to β-actin. Data are presented as the mean ± standard deviation (^*^P<0.05). BANCR, BRAF-activated long non-coding RNA; N, normal tissue; T, tumorous tissue.

To investigate the role of BANCR in PTC development, BANCR RNA expression levels in PTC tissue samples were examined first. The RT-PCR results showed that BANCR expression was significantly higher in five out of six of the tumor tissues compared with the adjacent normal tissues. Additionally, BANCR expression in the PTC IHH-4 cell line was upregulated compared with the mean expression level of the adjacent normal tissues (P<0.05; Fig. 1).

### BANCR-knockdown inhibits proliferation and increases apoptosis of PTC IHH-4 cells

**Figure 2:**
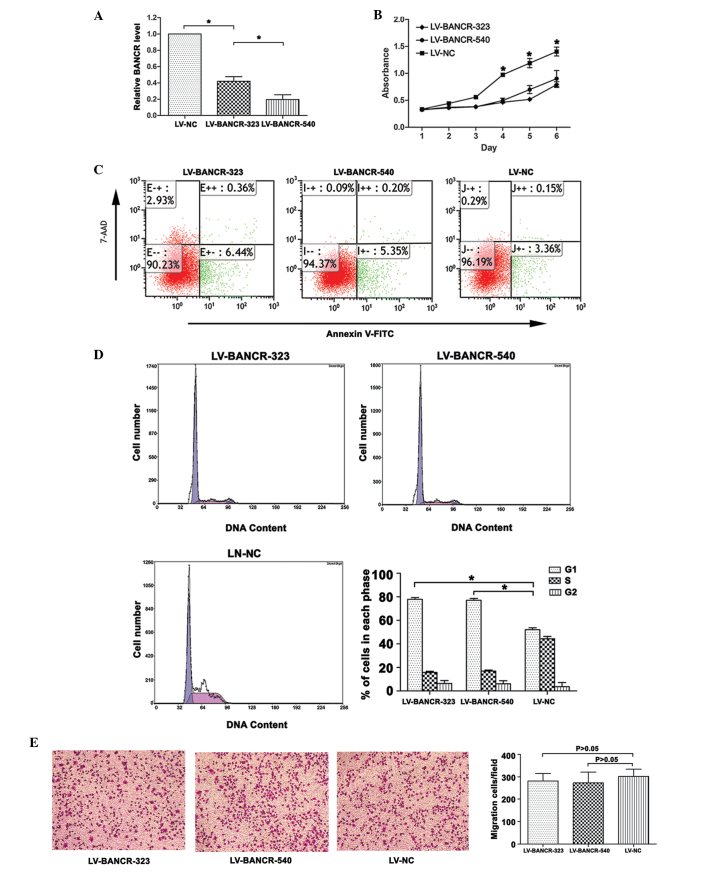
BANCR-knockdown inhibits cell proliferation and increases apoptosis in papillary thyroid carcinoma IHH-4 cells. (A) Following treatment with LV-BANCR-323 and LV-BANCR-540, BANCR expression in the IHH-4 cells was downregulated compared with the cells treated with LV-NC. (B) Cell Counting Kit-8 assays showed that BANCR-knockdown inhibited the proliferation of the IHH-4 cells. (C) Knockdown of BANCR induced apoptosis, which was detected by flow cytometry. The results are presented as the percentage of apoptotic cells in the total number of counted cells. (D) BANCR-knockdown resulted in an increase in the cell population in the G_1_ phase. The data represent one of at least three independent experiments. (E) The Transwell migration assays showed that BANCR-knockdown had no significant effect on the migration of IHH-4 cells. The images represent at least three independent experiments. The graph indicates the number of migrated cells per field. The results are presented as the mean ± standard deviation (^*^P<0.05). BANCR, BRAF-activated long non-coding RNA; LV-BANCR-323, lentivirus containing shRNA-323; LV-BANCR-540, lentivirus containing short haipin (sh)RNA-540; LV-NC, lentivirus negative control; 7-AAD, 7-amino-actinomycin D; FITC, fluorescein isothiocyanate.

Following infection with LV-BANCR-323 and LV-BANCR-540, the BANCR expression level was significantly downregulated compared with the LV-NC (P<0.05; Fig. 2A). As shown in Fig. 2C, the knockdown of BANCR induced cell apoptosis. The results were presented as the percentage of apoptotic cells in the total number of counted cells. Signals from apoptotic cells were localized in the lower right quadrant (F=603.832, P<0.05). The CCK8 assays revealed that the knockdown of BANCR inhibited the proliferation of the IHH-4 cells (P<0.05; Fig. 2B). Further investigation into the role of BANCR in the regulation of cell proliferation revealed that the knockdown of BANCR resulted in an increase in the cell population in the G_1_ phase (P<0.05; Fig. 2D). The Transwell migration assays showed that BANCR-knockdown had no significant effect on the migration of the IHH-4 cells (P>0.05; Fig. 2E).

### Overexpression of BANCR increases autophagy activation in PTC IHH-4 cells

**Figure 3:**
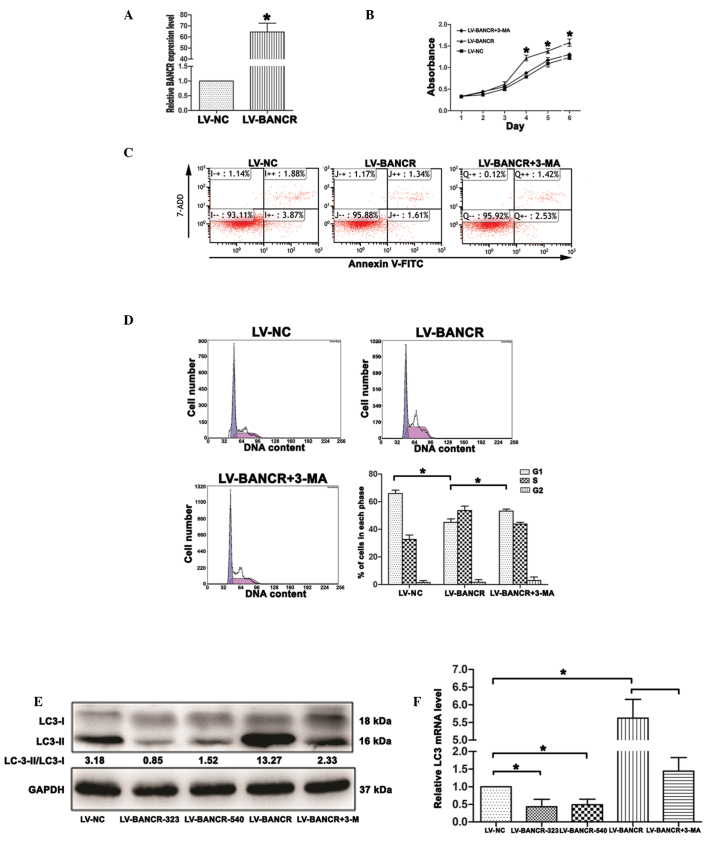
Overexpression of BANCR increases autophagy activation in papillary thyroid carcinoma IHH-4 cells.(A) Following treatment with LV-BANCR, BANCR expression in IHH-4 cells was upregulated compared with the cells treated with LV-NC. (B) Cell Counting Kit-8 assays showed that the overexpression of BANCR promoted cell growth, whereas the inhibition of autophagy inhibited the proliferation of the IHH-4 cells. (C) Overexpression of BANCR inhibited cell apoptosis, whereas autophagy inhibition increased apoptosis in the IHH-4 cells, which was detected by flow cytometry. (D) Overexpression of BANCR decreased the cell population in the G_1_ phase, whereas autophagy inhibition increased the cell population in the G_1_ phase. The data represent one of at least three independent experiments. (E) Western blotting results revealed that BANCR overexpression resulted in an increase in the ratio of LC3-II/LC3-I, while knockdown of BANCR and treatment with 3-MA decreased the ratio of LC3-II/LC3-I. (F) Reverse transcription polymerase chain reaction results showed that BANCR overexpression resulted in an increase in the LC3 mRNA level, while knockdown of BANCR and treatment with 3-MA decreased LC3 mRNA level. The results are presented as the mean ± standard deviation (^*^P<0.05). BANCR, BRAF-activated long non-coding RNA; LV-BANCR, lentivirus containing human full-length BANCR cDNA; LV-BANCR-323, LV containing short hairpin (sh)RNA-323; LV-BANCR-540, LV containing shRNA-540; LV-NC, LV negative control; 3-MA, 3-methyladenine; 7-AAD, 7-amino-actinomycin D; FITC, fluorescein isothiocyanate.

To explore the mechanism by which BANCR regulates cell proliferation, the present study investigated whether BANCR regulates cell autophagy. The IHH-4 cells were treated with LV-BANCR, with or without 3-methyladenine (3-MA), an inhibitor of autophagy. The results revealed that following infection with LV-BANCR, the BANCR expression level was significantly upregulated compared with the LV-NC (P<0.05; Fig. 3A). The overexpression of BANCR inhibited the apoptosis of the IHH-4 cells, promoted cell growth and decreased the cell population in the G_1_ phase, whereas autophagy inhibition increased cell apoptosis (F=167.557, P<0.05; Fig. 3C), inhibited cell growth (P<0.05; Fig. 3B) and increased G_1_ arrest (P<0.05; Fig. 3D) in the BANCR-overexpressed IHH-4 cells. The western blotting results demonstrated that BANCR overexpression resulted in an increase in the ratio of LC3-II/LC3-I, a marker for autophagy, while knockdown of BANCR and treatment with 3-MA decreased the ratio of LC3-II/LC3-I (P<0.05; Fig. 3E). The RT-PCR results indicated that the level of LC3 mRNA had increased in the BANCR-overexpressed cells, while it had decreased following BANCR-knockdown and in the 3-MA-treated cells (P<0.05; Fig. 3F).

## Discussion

Advances in molecular techniques have led to the identification of a novel type of gene regulators called lncRNA. These lncRNAs are >200 nucleotides and do not code for proteins. However, they can interact with proteins and can likely act as regulators of other genes (12). Although lncRNAs are not as well-characterized as small non-coding microRNAs, they play a critical role in the regulation of diverse cellular processes (13,14). In thyroid cancer, one such example of oncogenic lncRNA is papillary thyroid carcinoma susceptibility candidate 3 (PTCSC3). Using quantitative PCR, PTCSC3 expression was revealed to be strongly downregulated in thyroid tumor tissues, and it was demonstrated that the restoration of PTCSC3 expression in PTC cells inhibited cell growth and affected the expression of numerous genes (15). Another classic oncogenic lncRNA is non-coding RNA associated with the mitogen-activated protein (MAP) kinase pathway and growth arrest (NAMA), which is weakly expressed in thyroid cancer tissues. Knockdown of BRAF has been revealed to induce inhibition of the MAP kinase pathway, growth arrest and DNA damage in thyroid cancer cell lines (16).

BANCR is recurrently overexpressed in melanoma. In previous studies, shRNA-mediated knockdown of BANCR in melanoma cells was revealed to alter the expression levels of 88 genes, several of which are involved in cell migration and chemotaxis. BANCR depletion impaired the migration of the melanoma cells *in vitro* (5,6). Mutation of BRAF is hypothesized to be a putative prognostic marker for the aggressiveness of PTC (17). Based on these findings, it was hypothesized that BANCR could play a critical role in PTC. In the present study, it was found that BANCR expression levels were upregulated in five out of six PTC tumor tissues compared with their adjacent normal tissues. Although samples from only six patients were used in the present study and the results may not be entirely accurate due to type I or II errors, the present data suggest a possible oncogenic role of BANCR in several human cancers. Furthermore, *in vitro* examination of the potential role of BANCR in PTC IHH-4 cells demonstrated that the knockdown of BANCR in the IHH-4 cells was associated with the inhibition of proliferation and the promotion of apoptosis, but exhibited no significant effect on cell migration. This observation was in contrast with previous studies regarding the role of BANCR in regulating cell migration (5), thereby suggesting that the function of BANCR could be tissue-specific.

Autophagy is a self-degradative process through which the cytoplasmic materials within the lysosome are degraded. The process acts as a dynamic system that provides the building blocks of a cell and the energy for cellular homeostasis and regeneration (18). Maddodi *et al* observed that the presence of high levels of mBRAF triggers the hyperactivation of extracellular-signal-regulated kinase (ERK), a senescence-like phenotype and initiates autophagy through the inhibition of mammalian target of rapamycin complex signaling (11). BANCR was considered to play a role in controlling cell proliferation by regulating autophagy activation, although there was no direct evidence to support this hypothesis. In the present study, it was demonstrated that the overexpression of BANCR induced autophagy activation, whereas BANCR-knockdown decreased autophagy activation in the PTC IHH-4 cells. Autophagy activation was evaluated by observing the ratio of LC3-II/LC3-I. Overexpression of BANCR inhibited the apoptosis of the IHH-4 cells, promoted cell growth and decreased the cell population in the G_1_ phase; all these effects could be suppressed by 3-MA, an inhibitor of autophagy. These findings suggest that BANCR may increase PTC cell proliferation by activating autophagy.

To the best of our knowledge, this is the first study to report that BANCR is highly expressed in PTC and that BANCR is likely to be a useful biomarker of this disease. Additionally, the fact that BANCR increases PTC cell proliferation by activating autophagy adds to our understanding of the molecular mechanisms governing BANCR. Significantly, BANCR could be used as a potential molecular target to treat human PTC.
